# Analysis and Design of Social Presence in a Computer-Mediated Communication System

**DOI:** 10.3389/fpsyg.2021.641927

**Published:** 2021-05-24

**Authors:** Hiroki Kojima, Dominique Chen, Mizuki Oka, Takashi Ikegami

**Affiliations:** ^1^Graduate School of Arts and Sciences, University of Tokyo, Tokyo, Japan; ^2^Faculty of Letters and Science, Waseda University, Tokyo, Japan; ^3^Graduate School of Science and Technology, University of Tsukuba, Ibaraki, Japan

**Keywords:** social interaction, dyadic interaction, phone call, textchat, transfer entropy, social presence

## Abstract

Social presence, or the subjective experience of being present with another existing person, varies with the interaction medium. In general, social presence research has mainly focused on uni-directional aspects of each exchanged message, not on bidirectional interactions. Our primary purpose is to introduce such bidirectional evaluation by quantifying the degree of social presence with a few statistical measures. To this end, we developed a software called “TypeTrace” that records all keystrokes of online chat interactants and reenacts their typing actions and analyzed the results from different chat conditions, mainly focusing on the characterization of bi-directional interactions. We also compared the chat interaction patterns with the patterns from phone call datasets to investigate the difference of live communication in different media. The hypothesis of the experiment was that either richness or concurrency of communication is important for organizing social presence. Richness is defined by the variety of information at a time in communication and the concurrency is the number of temporal thread being processed at the same time. Our results show that when we merely increase the richness of information by presenting the typing process, the cognition of others' presence does not significantly increase. However, when the information concurrency is augmented by introducing the transmission of realtime text, we found that the transfer entropy between the interactants becomes considerably higher, and the social presence and emotional arousal, intimacy increased. High transfer entropy was also observed in the phone call dataset. This result shows that the mere augmentation of information richness does not necessarily lead to increased social presence, and concurrent communication is another critical factor for fostering vivid conversation in digital environments.

## 1. Introduction

Conversations are central to our social lives. In Face-to-face (FtF) circumstances, social interaction includes not just the exchange of verbal sentences, but also interactions with non-verbal means such as body gestures, vocal cues, temporal structures in speech like turn-taking, facial expressions, and gaze exchanges. It is known that the medium of communication affects, among other aspects of social interaction, affiliative behaviors, and the resulting outcomes (Sprecher, 2014).

Modern societies have become inundated by computer-mediated communication (CMC) systems. Since the early introduction of personal computers in the 1980s until the universal dissemination of smartphones in the 2010s, we have experienced a drastic influx of new CMCs. The lineage of CMC has diversified since the age of simple text-based chats, with the burgeoning of audiovisual teleconferencing systems and virtual, augmented, and mixed reality devices.

Nevertheless, text-based CMC remains one of the dominant communication channels of today. For instance, as of 2019, among the 3.2 billion people worldwide who own a smartphone (Statista, 2020), 1.6 billion use WhatsApp, 1.3 billion use Facebook Messenger, and 1.1 billion use WeChat (Clement, 2019) (all monthly active users). By contrast, in 2019, <10 million people worldwide owned a virtual reality device (Statista, 2018). All of these messenger applications include rich media functions such as making online video or audio calls and sending high-resolution images or audio files, but text-based chats also remain widely used among its users, albeit the exact statistic is unknown.

Short et al. (1976) initially introduced the term “social presence” in the context of telecommunication and conceptualized the ability of communication media to transmit social cues. Social cues consist of both verbal and non-verbal information, such as facial expressions, gestures, and physical appearance, and they serve to construct the “sense of being with another” (Biocca et al., 2003). Short et al. (1976) considered these cues the foundation of intimacy (feeling of connectedness to the partner) and immediacy (psychological distance to the partner). In the early period of research, social presence was viewed as a variable depending on the quality of media. In short, telecommunication generally was regarded as a lesser communication channel compared to the FtF communication because of its inability to transfer non-verbal cues.

This line of research continued to gain support in the 1980s, where Daft and Lengel conducted seminal analyses of the richness of information and media (Daft and Lengel, 1984, 1986). However, in the 1990s, Walther argued in opposition to media richness theories, stating that if enough time is spent on CMC, interactants can achieve a level of interpersonal relationship as high as FtF communications (Walther, 1992). Walther later argued that communication means specific to CMC could even create a more robust social bond compared to FtF (Walther, 1996) because it stimulates more self-disclosure than FtF and thus can lead to higher social attraction. This school of thought, called the social information processing theory (SIPT) (Walther, 2015), has helped researchers to transcend the simple dichotomy of rich and poor media and to scrutinize the social phenomena in CMCs more in-depth.

Since then, theoretical discussions of social presence concerning CMC faced the need to reconcile technologically mediated social interaction with unmediated interaction (Biocca et al., 2003). In remote learning environments, social presence has been measured to predict participants' learning satisfaction (Gunawardena and Zittle, 1997), and comparisons of different types of CMC have been analyzed (Tu, 2002). Antheunis et al. (2010) conducted a thorough quantitative analysis of an Social Networking Service (SNS) based on a hypothesized model of social presence theories and found supportive evidence of SIPT. A recent systematic review of mediated social presence research by Oh et al. (2018) marshaled different previously studied predictors of social presence, including new media such as virtual and augmented reality systems, while pointing out that social presence does not always lead to positive outcomes. The relationship between social presence and the valence of communication is yet to be further elaborated.

Moreover, the dominant trend in social presence research so far has been to treat only the change in the characteristics of each message, which is uni-directional. On the other hand, in the field of social cognition, researchers have argued that bi-directional interaction plays a central role in understanding dyadic interactions (Schilbach et al., 2013; Gallotti et al., 2017; Redcay and Schilbach, 2019).

In this study, we investigated these relationships by recording and analyzing the dyadic bi-directional interaction of CMC. We particularly focused on the temporal dynamics of interaction and each interlocutor's response during several types of text chat systems. In order to find evidence for potential factors that contribute to the generation of social presence in a dyadic CMC setup, we formulated the following two questions and designed our series of experiments accordingly.

First, how does the increase of informational richness affect interactions in CMC? The pre-SIPT line of theories predicted that the lack of social cues such as facial expressions would decrease social presence. However, neither SIPT nor later research rigorously measured such richness of information in text-based CMC. Secondly, how does the concurrency of information exchange between the interactants influence the dynamics of a CMC interaction? Past social presence research often mixed synchronous and asynchronous CMC such as chat, e-mail, and teleconferencing. In our study, we specialized in synchronous text chat in order to observe results varying on the difference of information concurrency. We employ transfer entropy to measure such degree of information concurrency.

Our analysis of keystroke dynamics focused on the coupling between the two subjects of text chat. To capture the bi-directional aspect of the text chat, analysis of time-series data of dyadic interaction is required. In this direction, some studies characterized temporal dynamics using some measures such as recurrence quantification analysis (Fusaroli and Tylén, 2016) and the Allan factor (Kello et al., 2017).

In this study, we used transfer entropy, which is a measure in information theory used to detect information flow between two time-series data (Schreiber, 2000). In our previous research, we analyzed the dyadic interactions in perceptual crossing experiment, which consisted of a minimal CMC that only involved a vibration device and a computer mouse, using the local form of transfer entropy (Lizier et al., 2008), and we found that passive information flow was related to the feeling of the presence of the others (Kojima et al., 2017).

We measured changes in the amount of transfer entropy between the four conditions of our experiment and also in relation with the phone call data set.

## 2. Methods

### 2.1. TypeTrace Messenger

TypeTrace is a software that records the entire typing processes of writing and replays it by varying the font size as a function of writing speed of each letter (i.e., the font size becomes larger when there is a slower writing speed). The software has also been used for a quantitative analysis of a professional creative writer's process of writing a new novel (Kudo et al., 2015). TypeTrace software has been demonstrated at several art exhibitions (e.g., Aichi Triennale 2019 exhibition).

We here developed a new TypeTrace Messenger (TT Messenger) based on the previous versions of TypeTrace. TT Messenger is a Web application that enables users to take part in dyadic chat online on PC browsers. We use Google Firebase for the backend system, and the software runs on modern Chrome browsers. We wrote the software in JavaScript and recorded typing data in the JSON format.

TT Messenger records all key typing during a chat session and is capable of precisely reenacting each typing action. This playback includes all the processes of typing, such as pauses, corrections, and deletions.

**Figure 1:**
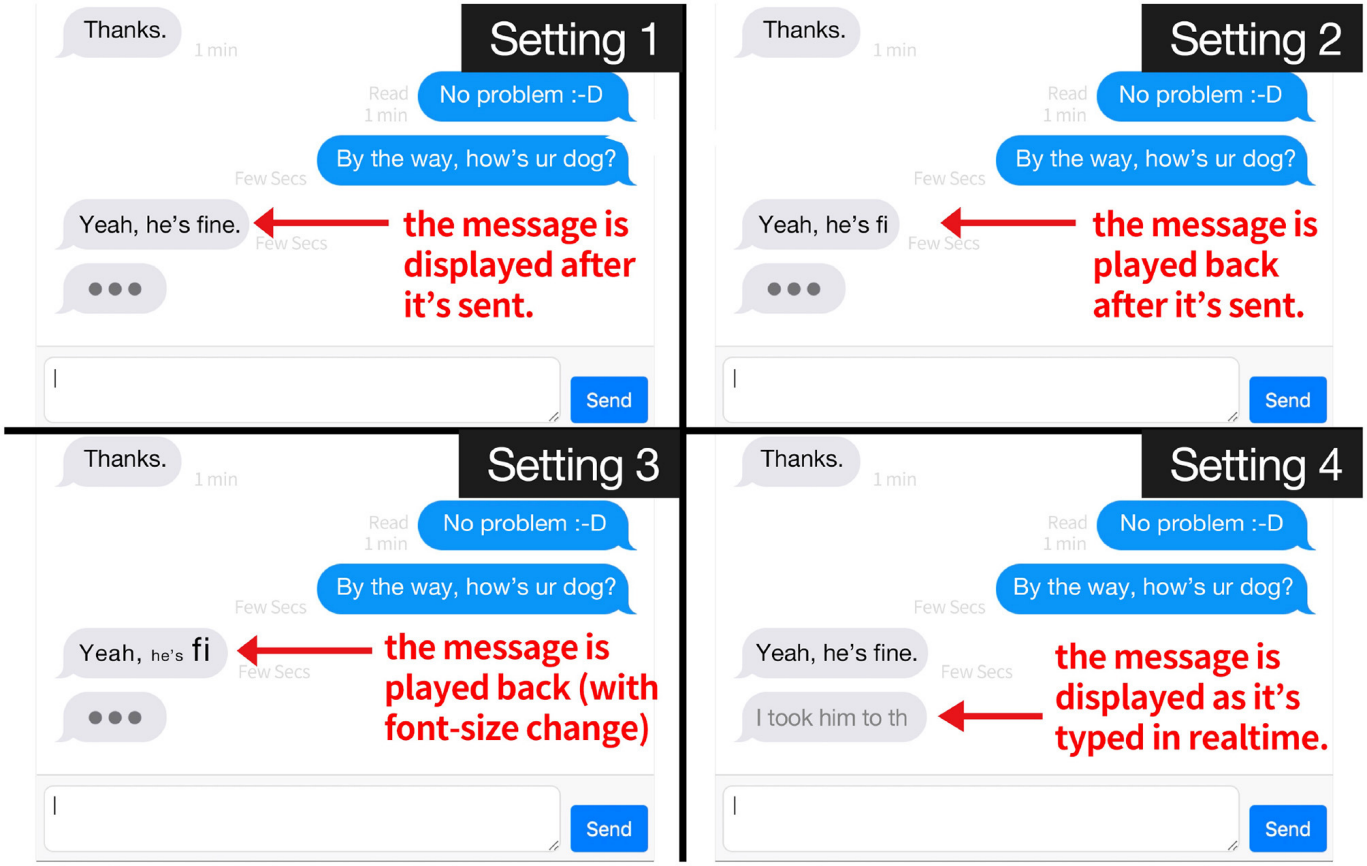
Screenshots of the four different conditions of our chat system, TypeTrace Messenger. The actual chats in our experiments were conducted in Japanese, but we created this figure with English texts for explanation purposes. In condition 1, the messages are displayed statically, which corresponds to a regular online chat system. In condition 2, the whole process of typing is dynamically displayed, not just the static messages. In conditions 2 and 3, the font size of the messages changed according to the time to type that message. In condition 4, the content the subjects are typing is simultaneously shown in the other's display. The video is provided as Supplementary Video 1.

TT Messenger has four different conditions (Figure 1):

1. It looks like a regular online chat system. Before the partner sends a message, the recipient can only see a dotted line, which shows that the partner is typing something. When the partner sends the message, the recipient can see it as a static text.
2. The recipient sees the partner's message in a dynamic playback (dynamically presenting the playback of the other's text message typing) as soon as she receives it. Therefore, recipient has to wait until the playback finishes in order to see the resulting final message. We designed this setup to consider our first question on the richness of information exchanged between interactants.
3. Just like in the second condition case, the messages play back as soon as they are sent, but with an additional visual effect. The software records the duration taken to type every word and changes each word's respective font sizes as it plays them back. For instance, when a user takes three seconds to finish typing a word, that word would appear with a bigger font size than the previous word that took only one second to type. We added this effect in order to visualize the rhythm of the typing. We hypothesized that this additional social cue would have a comparable effect with facial expressions and body gestures in FtF communication. We expected the results from this condition would shed light on our first question about information richness.
4. The text chat becomes concurrent, and it works in real time. As soon as the partner starts typing, the process is transmitted to the recipient's screen in real time, even without the partner sending the text. The partner can send the message at any moment, but they do not have to. The two parties can simultaneously type, and each other's messages are displayed at the same time. We designed the fourth condition to examine our second question on information concurrency.

In the following sections, we explain how we used these four configurations for our experiment.

### 2.2. Participants

Participants were healthy volunteers recruited from acquaintances at Waseda University (*N* = 18). They were all Japanese nationals; 11 were female, and the median age was 22 years old. All pairs were already acquainted before the experiments. We asked the participants about their habit of text chat systems by asking how often they used some kinds of text chat systems from seldom, sometimes, frequently or every day and 15 subjects answered they used text chats on daily basis, 2 subjects answered they used frequently, and 1 subject answered seldom. The study protocol was approved by the local ethics research committee of Waseda University (Ethics Review Procedures concerning Research with Human Subjects; Application Number:2018-273; Approved on 25th of January, 2019), and the methods were carried out according to the ethics committee guidelines and regulations. All of the participants gave their written informed consent before taking part in the study.

### 2.3. Experimental Procedures

Two participants are placed in different rooms. Each are provided with a laptop PC, and we asked them to freely converse with each other through TT Messenger. We did not set a theme for the conversations. For each trial, we asked the pairs to converse for 10 min and to answer questionnaires after that. The experiments consisted of two rounds of four sessions, and each session included every condition (1–4) of TT Messenger in random order.

**Figure 2:**
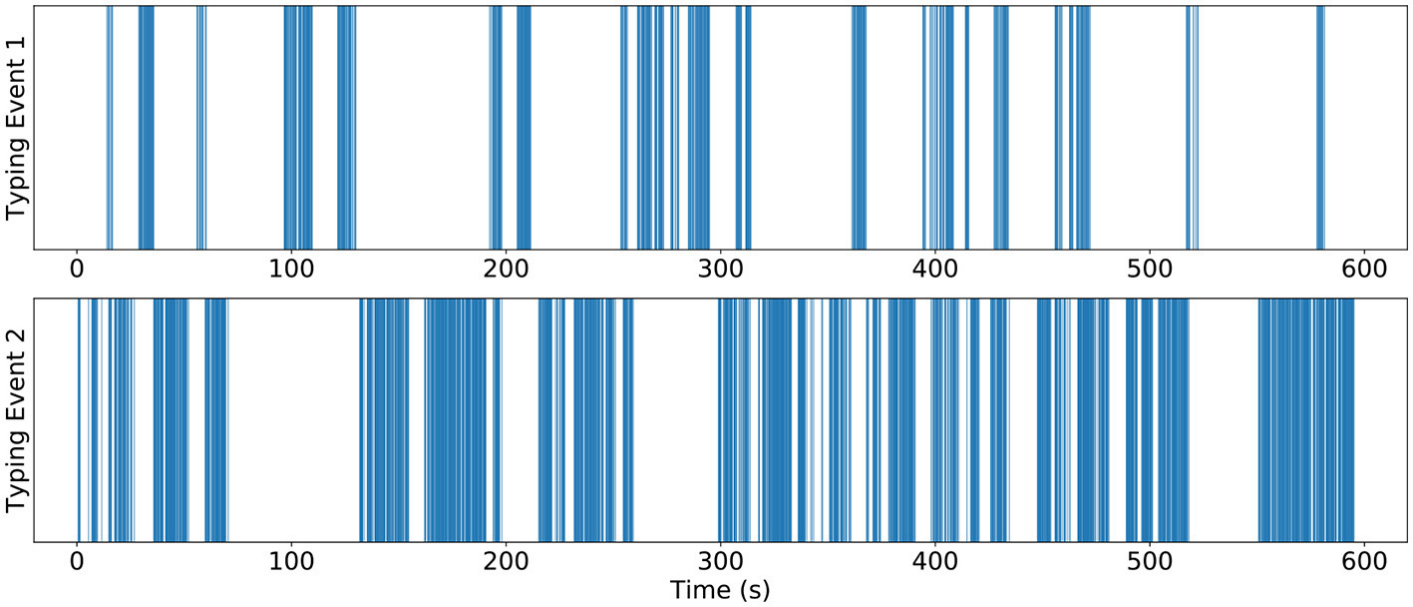
Example of the keystroke data from two subjects in one trial. Each vertical line corresponds to one keystroke event.

During each session, we recorded the keystroke events, galvanic skin response (GSR), and facial expressions. An example of the keystroke timeseries data is shown in Figure 2.

### 2.4. Subjective Reports

We used a five-point Likert scale to estimate the subjective rating of the degree of nervousness, enjoyment, closeness, presence of the other, and time delay. Actual questionnaire items in Japanese and English translations are listed below.

- I got nervous during the conversation. (相手との会話に緊張した)
- I had an enjoyable conversation. (会話がはずんだ)
- I felt close to the partner. (相手との距離が近く感じられた)
- I strongly felt the presence of the partner. (相手の存在感を強く感じた)
- I felt the time delay when exchanging messages. (メッセージのやり取りに時間がかかったと感じた)

We also asked each subject to report the Inclusion of Other in the Self (IOS) scale before the experiment and after each trial. The IOS scale has been used to measure the subjective closeness to others and is known to correlate well to other subjective markers of interpersonal closeness (Aron et al., 1992).

### 2.5. Measurements

During the experiments, we recorded galvanic skin response (GSR) by Shimmer GSR sensors and facial expressions by the web camera mounted on the computer, which were later analyzed by OpenFace (Baltrušaitis et al., 2016) to extract action units (AU). We measured GSR of one subject from the pair and switched to the other subject on the second round of experiments. Facial expressions were simultaneously recorded from both subjects. Keystroke events are collected through TT Messenger.

### 2.6. Transfer Entropy

Transfer entropy (Schreiber, 2000) from time series process *Y* to *X* is formulated using conditional mutual information as

$$T_{Y\to X}=I(Y_{n+1}^{\left(l\right)};X_{n+1}|X_{n}^{\left(k\right)}),$$

where $X_{n}^{\left(k\right)}=\left\{X_{n-k+1},...,X_{n}\right\}$, $Y_{n}^{\left(l\right)}=\left\{Y_{n-l+1},...,Y_{n}\right\}$ (*k*: target history length, *l*: source history length).

Effective transfer entropy (Marschinski and Kantz, 2002) is calculated by subtracting the mean value of null distribution of transfer entropy, which is constructed by calculating the transfer entropy with a resampled surrogate source time series, from the original transfer entropy. We calculated the effective transfer entropy between subjects' keystroke event time series (or phoneme event time series for the phone call data) downsampled to 100 ms windows. We used JIDT (Lizier, 2014) for the calculation, and we set *k* = *l* = 2.

### 2.7. Phone Call Dataset

In order to compare the keystroke dynamics with the dyadic dynamics that have different modalities, we used a conversation corpus, CallFriend (Yaeger-Dror, 2004), which consists of telephone conversations data in Japanese. From this corpus, we used the audio data of 41 conversations with age information (file IDs were ja_1722, ja_4044, ja_4164, ja_4222, ja_4261, ja_4573, ja_4608, ja_4905, ja_6149, ja_6166, ja_6167, ja_6186, ja_6221, ja_6228, ja_6264, ja_6277, ja_6354, ja_6414, ja_6416, ja_6422, ja_6434, ja_6463, ja_6465, ja_6484, ja_6490, ja_6525, ja_6587, ja_6616, ja_6630, ja_6632, ja_6666, ja_6688, ja_6698, ja_6700, ja_6707, ja_6716, ja_6717, ja_6738, ja_6739, ja_6742, ja_6759). The median age was 28 years old, and we used the first 10 minutes from each audio file.

For the extraction of phoneme events from audio data, we applied the phoneme segmentation method by Ziółko et al. (2006). This method is based on a six-level discrete wavelet transform (DWT) analysis, and it detects the boundary of phonemes as the time of rapid change in each subband power. We used the sym6 wavelet and set a minimal threshold of subband DWT power, *p*_min_, to 0.005. The other parameters were kept the same as in the original paper.

We used the boundaries of the phoneme segmentation as the phoneme events' time series, comparable to the keystroke events for TT Messenger data, and applied the same analysis to the event sequences.

## 3. Results

Below, we report the results from subjective reports, physiological markers, and keystroke dynamics, and compare among different conditions of the chat system and telephone conversation data. If not otherwise stated, we used the Friedman test for statistical testing and the Nemenyi test for *post-hoc* testing.

### 3.1. Subjective Reports

**Figure 3:**
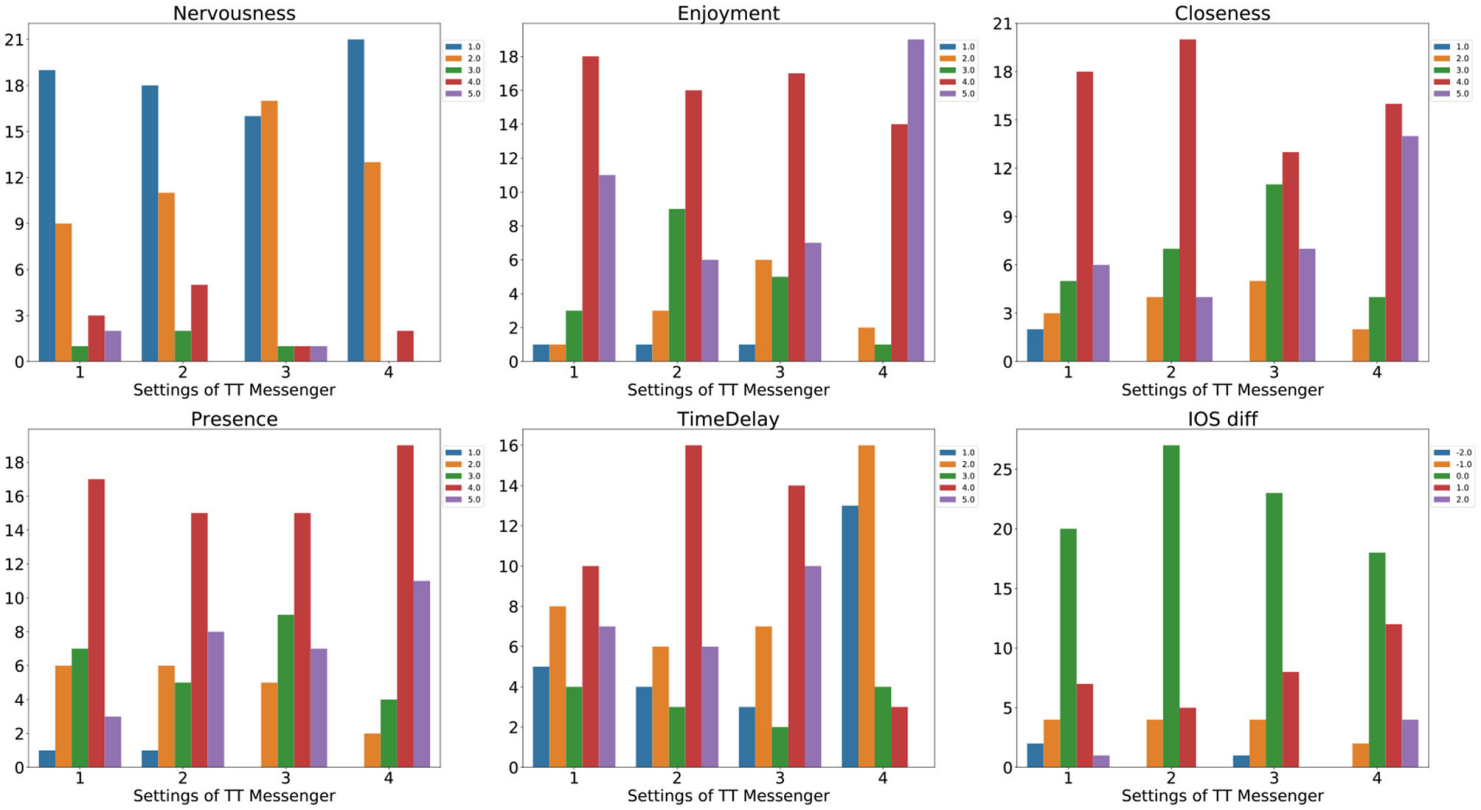
Histograms of subjective ratings in each condition of TT Messenger for Nervousness, Enjoyment, Closeness, Presence, Time Delay, and IOS Change (from top left to bottom right). Enjoyment was significantly higher in condition 4 than 2 (*p* < 0.05), and 3 (*p* < 0.05), Closeness was significantly higher in condition 4 than 3 (*p* < 0.05), Presence was significantly higher in condition 4 than 1(*p* < 0.05), and Time Delay was significantly smaller in condition 4 than 1 (*p* < 0.05), 2 (*p* < 0.05), and 3 (*p* < 0.05). No significant difference was found in the rating of Nervousness (*p* = 0.4) and IOS (*p* = 0.06).

First, we investigated the subjective reports after each session. The histogram of ratings for each item in different conditions of TT Messenger is shown in Figure 3.

We found that in condition 4, the rating of Enjoyment was significantly higher than it was in condition 2 (*p* < 0.05) and condition 3 (*p* < 0.05), the rating of Closeness was significantly higher than it was in condition 3 (*p* < 0.05), and the rating of Presence was significantly higher than it was in condition 1 (*p* < 0.05). The rating of Time delay was significantly smaller in condition 4 than it was in conditions 1 (*p* < 0.05), 2 (*p* < 0.05), and 3 (*p* < 0.05). No significant difference was found in the rating of Nervousness.

Also, we investigated the change in IOS before and after each trial. We found that the percentage of positive change was 22, 14, 22, and 44% in conditions 1, 2, 3, and 4, respectively, but there was no significant difference among these conditions (*p* = 0.06).

### 3.2. Physiological Markers

In order to confirm the result from subjective reports, we also recorded physiological markers. Here, we used GSR and facial expressions extracted by OpenFace (Baltrušaitis et al., 2016) to recognize the emotional state of the subjects.

#### 3.2.1. GSR

We recorded GSR, which is related to states of arousal (Dawson et al., 2017), during each trial. We calculated the median value from the time series and subtracted the initial value to characterize the amount of increase of GSR during each trial.

**Figure 4:**
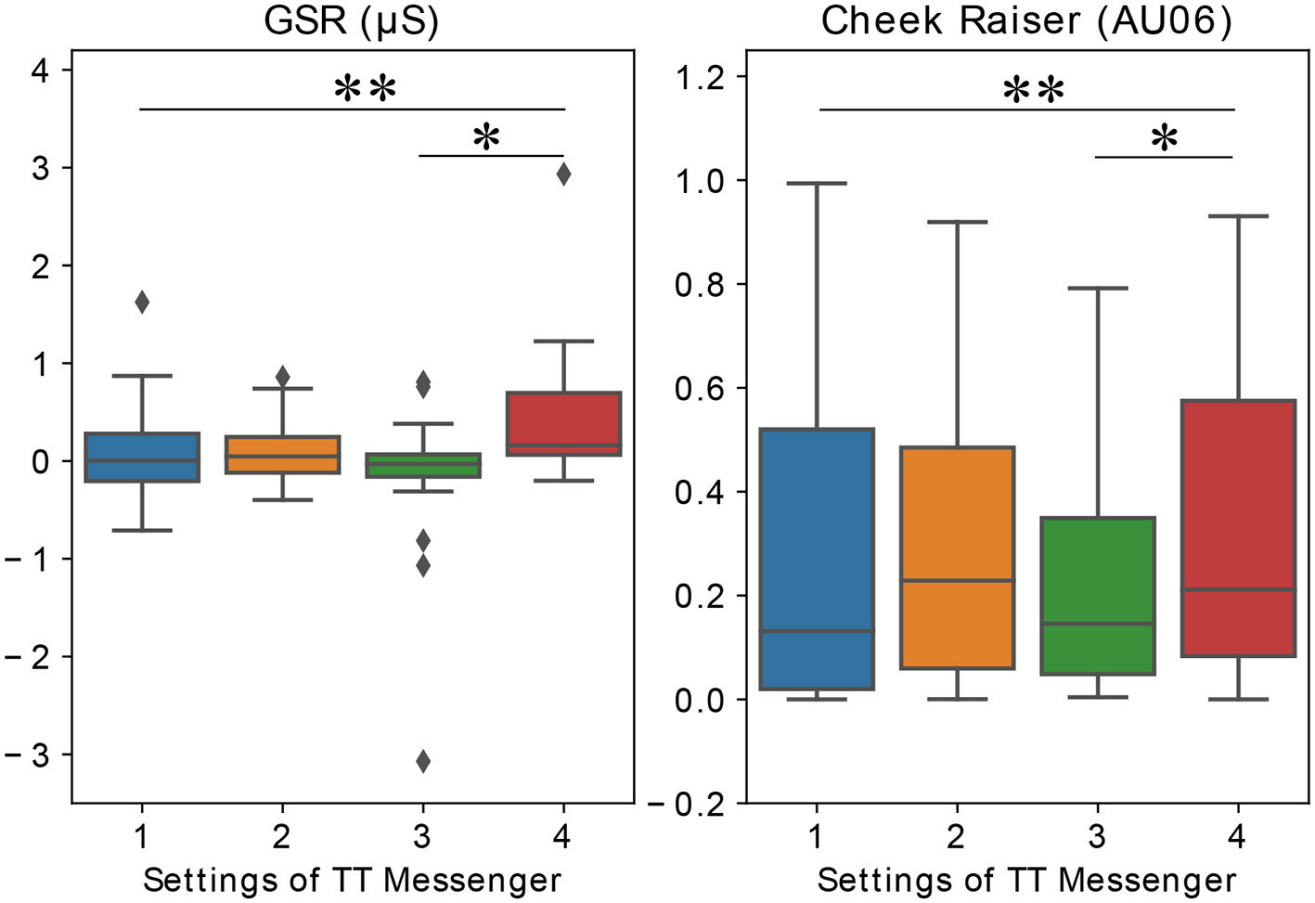
Results of physiological markers (median, IQr, and 95% CrI). **(Left)** The median value of GSR during each trial for different conditions of TT Messenger. **(Right)** The median value of AU6 (Cheek Raiser) during each trial for different conditions of TT Messenger (*p < 0.05, **p < 0.01).

The median values of GSR from all trials were 4.7 × 10^−3^μS, 4.7 × 10^−2^μS, −3.0 × 10^−3^μS, 0.16μS for conditions 1, 2, 3, 4, respectively (Figure 4). Also, we found the GSR values from condition 4 were significantly higher than the values from conditions 1 (*p* < 0.005) and 3 (*p* < 0.05).

#### 3.2.2. Cheek Raiser (AU6)

We recorded facial expressions with web cameras during each trial and analyzed using OpenFace (Baltrušaitis et al., 2016). OpenFace extracted the elementary facial motion unit, action unit (AU). We used AU6 (cheek raiser), which is related to the feeling of happiness (Ekman, 1997; Sato et al., 2019).

The median values of AU6 from all trials were 0.13, 0.23, 0.15, 0.21 for conditions 1, 2, 3, 4, respectively (Figure 4). Also, we found that the values of AU6 from condition 4 were significantly higher than the values from condition 1 (*p* < 0.005) and 3 (*p* < 0.05).

### 3.3. Keystroke Dynamics

Dyadic conversations are characterized by synchrony of utterances and the turn-taking patterns. We quantify them in each of the four conditions to study the differences.

#### 3.3.1. Synchronization in Typing Patterns

In order to quantify the synchrony in typing patterns, we used two measures, Jensen-Shannon divergence (JS-divergence) between histograms of inter keystroke intervals (IKSIs) and correlation coefficient in the medians of IKSIs.

**Figure 5:**
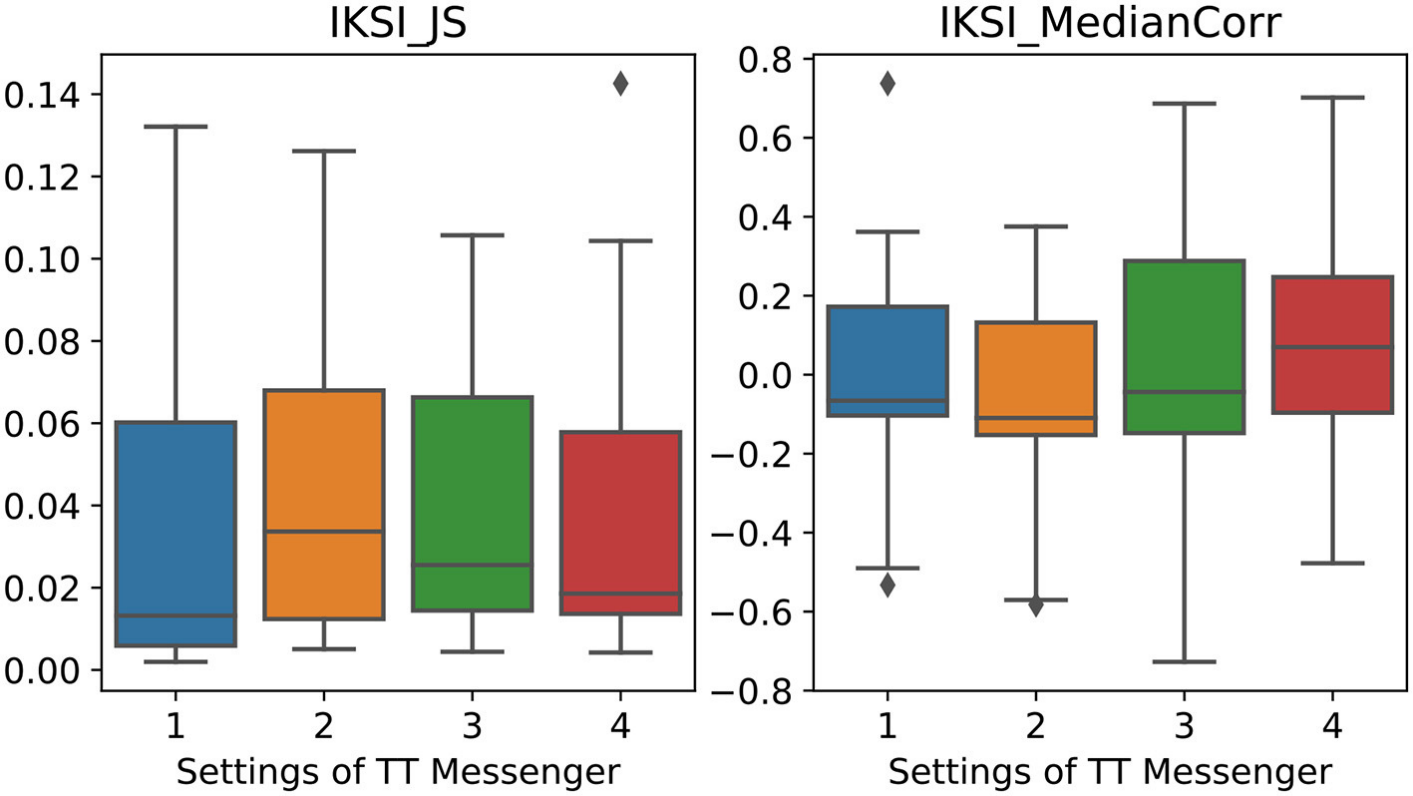
Two synchronization measures between subjects' keystroke event timeseries (median, IQr, and 95% CrI). **(Left)** JS-divergence between two subjects' IKSIs histograms of each trial for different TT Messenger conditions. **(Right)** Correlation coefficient between IKSIs among 1 min segments in different TT Messenger conditions.

First, we used JS-divergence of the IKSI histograms between subjects in pairs to measure the dissimilarity in typing patterns in each trial and compare them among different TT Messenger conditions. The median values of JS-divergence were 0.015, 0.034, 0.026, and 0.018 for conditions 1, 2, 3, and 4, respectively (Figure 5), and there was no significant difference among these conditions(*p* = 0.5).

Secondly, in order to measure the degree of synchronization in typing speed during each trial, we split each trial into 1-min windows, calculated the median values of IKSIs of each subject for all 10 windows, and calculated the correlation coefficient of the median values between the two subjects. The median values of results from all pairs were −0.07, −0.1, −0.04, and 0.07 for conditions 1, 2, 3, and 4, respectively (Figure 5), and there was no significant difference among these conditions (*p* = 0.4).

#### 3.3.2. Pattern in Turns

**Figure 6:**
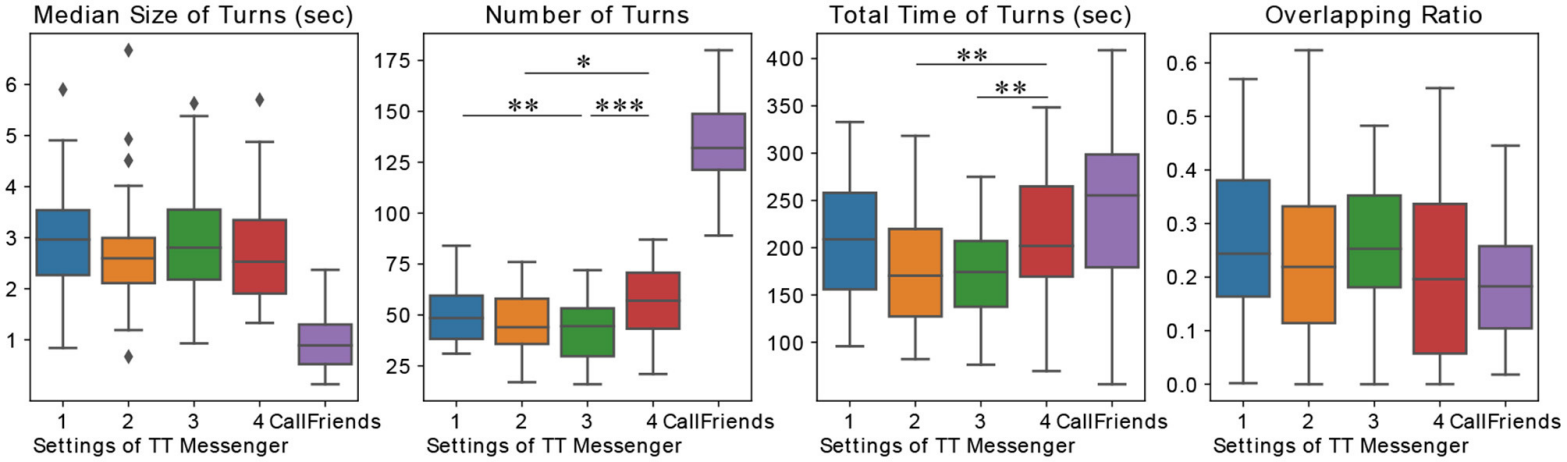
Four measures to characterize the structure of turns, which are identified from chunking keystroke event timeseries, median size of turns (sec), number of turns, total time of turns (sec), and overlapping ratio between dyads (median, IQr, and 95% CrI). Each measure was calculated using different TT Messenger conditions and phoneme timeseries data obtained from the telephone conversation dataset, using CallFriend (*p < 0.05, **p < 0.01, ***p < 0.001).

In order to characterize the global typing patterns, we analyzed the pattern in the chunk of keystroke events (which we call turns) as follows. We identified each turn by chunking a keystroke event within the threshold interval, which we set to 2 s. (The actual algorithm is described in section 2.) We used median size of turns (sec), number of turns, total time of turns (sec), and overlapping ratio between two subjects in each trial to characterize the turn structure (Figure 6).

We found no significant difference among the median size of turns (the median values were 3.0, 2.6, 2.8, and 2.5 s for conditions 1, 2, 3, and 4, respectively *p* = 0.9) and overlapping ratios (the median values were 0.24, 0.22, 0.25, and 0.20 for conditions 1, 2, 3, and 4, respectively, *p* = 0.2).

On the other hand, the median values of the number of turns were significantly different for those conditions. They were 48.5, 44.0, 44.5, and 57.0, for conditions 1, 2, 3, and 4, respectively, and the numbers in condition 4 were significantly higher than those in conditions 2 (*p* < 0.05) and 3 (*p* < 0.001). Also, the median values of total time for typing were 2.1 × 10^2^s, 1.7 × 10^2^s, 1.7 × 10^2^s, and 2.0 × 10^2^s for conditions 1, 2, 3, and 4, respectively. The numbers in condition 4 were significantly higher than those in conditions 2 (*p* < 0.01) and 3 (*p* < 0.01).

### 3.4. Information Flow Between Keystrokes of Partners

A second remarkable aspect of dyadic communication is the direct perception of the other's presence. We assume that when a subject's utterance is more driven by the other, the sense of presence increases. In such moment, the subject becomes less autonomous and more passive. The sense of passive awareness becomes the source of producing the presence of others (Kojima et al., 2017). This point will be revisited later.

We used effective transfer entropy (Schreiber, 2000; Marschinski and Kantz, 2002) to measure the information flow between subjects' keystroke events. We downsampled the keystroke event time series to a 100 ms window and calculated effective transfer entropy with *k* = *l* = 2.

**Figure 7:**
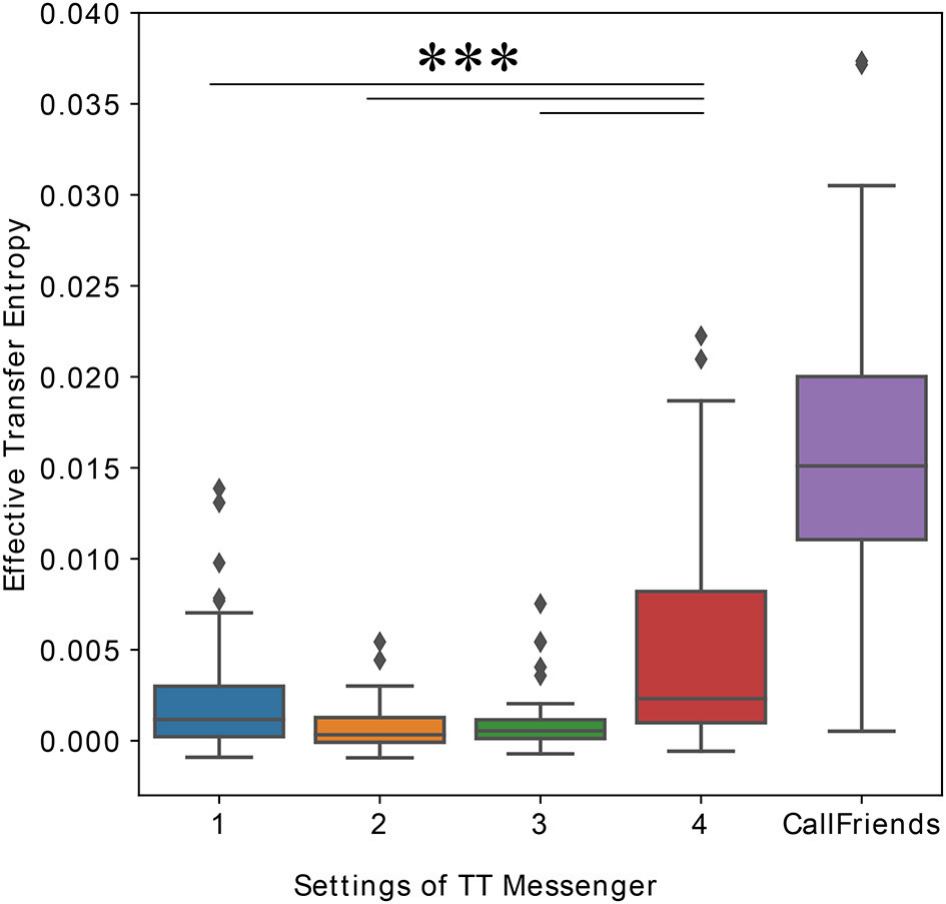
Effective transfer entropy between subjects' keystroke events timeseries data or phoneme events timeseries data from the telephone conversation data (median, IQr, and 95% CrI). Effective transfer entropy was calculated between two timeseries downsampled to 100 ms windows, and *k* = *l* = 2 (***p < 0.001).

The median values of effective transfer entropy in each condition were 1.2 × 10^−3^, 3.7 × 10^−4^, 5.5 × 10^−4^, and 2.3 × 10^−3^ for conditions 1, 2, 3, and 4, respectively (Figure 7), and the numbers in condition 4 were significantly higher than those in condition 1 (*p* < 0.001), condition 2 (*p* < 0.001), and condition 3 (*p* < 0.001).

### 3.5. Comparison to Phone Call Dynamics

So far, we have analyzed the chat data obtained from our chat system, TypeTrace Messenger. In order to compare these results with different types of dyadic interactions, we also used publicly distributed telephone conversation data from CallFriends corpus (Yaeger-Dror, 2004) and analyzed the data in the same way as we did to our chat data.

A phone call is not a CMC *per se*, but it is still omnipresent in modern societies and is a common feature included in many CMC applications. At the same time, although the modality differs radically between voice and text, a phone call resembles our experimental settings of text chat where participants are separated in different locations and converse without non-verbal social cues such as facial expressions and eye gazes. In both phone calls and text chats, participants spontaneously take turns, with overlaps in their utterances. However, we did not compare with FtF conversation because the structure of interaction differs even more substantially between FtF and text-chat.

CallFriends consists of sound data of actual telephone conversations and their scripts. We analyzed phone call sound data of 82 Japanese individuals (Yaeger-Dror, 2004). For pre-processing, we first extracted phoneme events from audio file (Ziółko et al., 2006) and analyzed these phoneme events' time series in the same manner as above.

First, we analyzed the turn structure of the telephone conversation (Figure 6). The median values of the median size of turns, number of turns, total time of turns, and overlapping ratio were 0.89, 132.0, 2.6 × 10^2^s, and 0.18, respectively. For statistical testing, we performed a Kruskal–Wallis test with a Mann–Whitney *U*-test as *post-hoc*, and we found that median size of turns was significantly smaller than that of our chat data with every condition (*p* < 0.001), and the number of turns and total time of turns were significantly longer than that of our chat data (*p* < 0.001, except between total time of turns in with condition 1 and the telephone conversation, *p* < 0.05, and condition 4 and the telephone conversation, *p* = 0.1).

Secondly, we calculated effective transfer entropy between phoneme events timeseries from dyads. We downsampled the phoneme event time series to a 100ms window and calculated effective transfer entropy with *k* = *l* = 2, in the same way as in the chat analysis. The median value of the effective transfer entropy of the phoneme events from a telephone conversation was 0.015 (Figure 6). For statistical testing, we performed a Kruskal–Wallis test with a Mann–Whitney *U*-test as *post-hoc* and found that the transfer entropy of the telephone conversation was significantly higher than the transfer entropy of our chat data with every condition (*p* < 0.001).

## 4. Discussion

With the aim to increase the social presence in our text chat system, we escalated the measures of richness and concurrency by introducing four different steps.

Richness of conversation designates the excess amount of information conveyed with communication. For example, in case of a dyadic conversation, the richness increases by introducing environmental sounds, bodily gestures, facial expressions, eye directions, and so forth. We formally introduced the richness of the communication in our experiment in a systematic way.

Concurrency signifies multiple events happening simultaneously. For example, while in a dyadic conversation, people often look away, unconsciously touch things, and some unexpected disturbances (e.g., coffee is served by a waiter or suddenly a dog barks) await. In this paper, TypeTrace emphasizes this concurrency effect.

First, we can increase information richness by presenting the playbacks of the typing process in TypeTrace chatting (in the case of conditions 2 and 3). Transfer entropy between the interactants becomes lower, and the cognition of the presence of others does not increase. We discuss the interpretation of this result below.

Secondly, we can increase the concurrency of interaction, namely the concurrency of information flow, by adding “redundant” elements to the main body messages (which is exemplified in the condition 4). Emotional arousal and intimacy increase as the result of the condition 4, and transfer entropy between the interactants becomes higher. We interpret the increase of transfer entropy from the other to self as the sign of increasing the sense of presence (e.g., Kojima et al., 2017). Together with the subjective reports, we affirm that the concurrency of information is an important factor for fostering vivid conversation in CMC.

As for the comparison to the phone call data set, transfer entropy of the phone conversation revealed to be close to that of condition 4. Additionally, the number of turns and the total time of turns are significantly greater in condition 4 when compared to conditions 2 and 3. And although we have only found a tendential increase of the number of turns in condition 4 than in condition 1, we argue that the increase of concurrency of condition 4 makes its dynamics closer to a phone call conversation.

In our past finding (Kojima et al., 2017), results from the perceptual crossing experiment (PCE) suggest that the feeling of the presence of the partner, or social presence, significantly correlates with the sense of being touched by the other (passive touch). This is supported by our analysis of the transfer entropy of the two interactants' inputs. A high transfer entropy from A to B means that the information that A possesses contributes more to determining the future states of B. Another way to put it is that B's actions are not self-determined, but are determined by A. We adapted this interpretation to the results of the calculations of this current study. Our subjective reports, physiological measurements, and informational analysis confirm that social presence correlates with intimacy (social attraction), immediacy (psychological distance), and interactivity among CMC participants. Our results also suggest that it is possible to augment the level of social presence evoked by a text-based CMC by increasing the concurrency of information flow between participants. Based on our results, we believe that transfer entropy can be a measure of the social presence in a CMC environment and could serve as an important design principle for such communication systems.

Our experiments gradually manipulated the granularity of the incoming partner's message. Our initial prediction was that the social presence could be augmented by showing the typing process of the received messages (condition 2) and the automatic changes of font sizes (condition 3). However, neither transfer entropy nor subjective reports were higher in these conditions than in the standard chat setting. We speculate the reason is because the typing playback itself causes a delay in synchronous chat communication. The receiver has to wait until the playback finishes to understand the message entirely. This time delay causes a non-negligible effect on the perception of social presence and transfer entropy. Indeed, scores of subjective reports and physiological data show that positive emotions in those circumstances were lower than in the standard chat. On a side note, if we experimented asynchronous chat in a longitudinal setup, the playback effect might have caused an increase in social presence at the moment the message is open. The playback effect might generate an illusory perception that the text is being typed in real-time. Further research is needed to prove this point.

Early researches of CMC argued that their lack of non-verbal cues lowers the social presence of their participants (Short et al., 1976; Daft and Lengel, 1984). Richness of information and media in CMC were considered the major predictor for satisfactory communication. Later, the Social Information Processing (SIP) theory (Walther, 2015) suggested that accustomed users find and use alternative cues specific to CMC systems in order develop interpersonal relationships, and rejected the idea that the quality of CMC is merely determined by the richness of the media involved (Walther, 1992). Since then, researchers have been pursuing the difference in the levels of social presence depending on the richness of medium involved in CMC (Oh et al., 2018), but some researches suggest that richness of media can sometime have negative impact on the communication (Dinakar et al., 2015).

We consider our current study contributes to the Social Presence literature, and more specifically, in relation with the field of Human-Computer Interaction (HCI), by introducing transfer entropy, an informationally quantitative measurement that is congruous with psychological reports and physiological markers. This results emphasizes the significance of information concurrency, which could be used for analyzing social presence in addition to the richness of media. Further research is needed to evaluate the impact of concurrency and social presence in a longitudinal setup with large number of subjects and different language like English, in order to understand its benefits and drawbacks on the mind of CMC users. Finally, the fact that our cognition of social presence and emotions are affected by the CMC system we use suggests both social responsibility and further possibility for designing better CMC systems to improve their users' well-being (Liu et al., 2019). We hope that the current research would lead to a more precise comprehension of the nature of social presence for designing systems that contribute to a more affective and inclusive CMC, especially in our time after the COVID-19 pandemic where the social impact of communication media is ever-growing globally.

## Data Availability Statement

The raw data supporting the conclusions of this article will be made available by the authors, without undue reservation.

## Ethics Statement

The studies involving human participants were reviewed and approved by the local ethics research committee of Waseda University. The patients/participants provided their written informed consent to participate in this study.

## Author Contributions

HK, TI, and DC conceived the experiment(s), analyzed the results, and wrote the manuscript. HK and DC conducted the experiment(s). MO helped with the writing and contributed to the analysis. All authors reviewed the manuscript.

## Conflict of Interest

The authors declare that the research was conducted in the absence of any commercial or financial relationships that could be construed as a potential conflict of interest.

## Supplementary Material

Supplementary Video 1 The showcase of four different settings of our chat system, TypeTrace Messenger.
